# Direct observations of ice seasonality reveal changes in climate over the past 320–570 years

**DOI:** 10.1038/srep25061

**Published:** 2016-04-26

**Authors:** Sapna Sharma, John J. Magnuson, Ryan D. Batt, Luke A. Winslow, Johanna Korhonen, Yasuyuki Aono

**Affiliations:** 1 Department of Biology, York University, Toronto, Ontario, M3P1J3, Canada; 2 Center for Limnology, University of Wisconsin-Madison, Madison, Wisconsin, 53706, USA; 3 Department of Ecology, Evolution, and Natural Resources, Rutgers University, New Brunswick, New Jersey, 08901, USA; 4 Center for Integrated Data Analytics, United States Geological Survey, 8505 Research Way, Middleton, Wisconsin, 53562; 5 Freshwater Centre, Finnish Environment Institute, Helsinki, FI-00260, Finland; 6 Graduate School of Life and Environmental Sciences, Osaka Prefecture University, Osaka, 599-8531, Japan

## Abstract

Lake and river ice seasonality (dates of ice freeze and breakup) responds sensitively to climatic change and variability. We analyzed climate-related changes using direct human observations of ice freeze dates (1443–2014) for Lake Suwa, Japan, and of ice breakup dates (1693–2013) for Torne River, Finland. We found a rich array of changes in ice seasonality of two inland waters from geographically distant regions: namely a shift towards later ice formation for Suwa and earlier spring melt for Torne, increasing frequencies of years with warm extremes, changing inter-annual variability, waning of dominant inter-decadal quasi-periodic dynamics, and stronger correlations of ice seasonality with atmospheric CO_2_ concentration and air temperature after the start of the Industrial Revolution. Although local factors, including human population growth, land use change, and water management influence Suwa and Torne, the general patterns of ice seasonality are similar for both systems, suggesting that global processes including climate change and variability are driving the long-term changes in ice seasonality.

Quantitative, direct annual observations by humans of climatic variables starting before 1840s and the Industrial Revolution are rare. Most studies of long-term climate have used time series of human observations that begin near the start of the Industrial Revolution, and rely on paleo-chronologies to assess climate conditions prior to the start of the Industrial Revolution^1^,^2^,^3^. Importantly, our paper is based on ice seasonality of inland waters collected directly by humans extending centuries prior to the start of the Industrial Revolution.

Lake and river ice seasonality (dates of ice freeze and breakup) responds sensitively to climatic change and variability^4^,^5^,^6^,^7^. Inland waters around the Northern Hemisphere since the start of the Industrial Revolution reveal strong trends of later freeze, earlier breakup, and shorter ice-cover duration^4^,^5^,^6^,^7^. Ice seasonality in North America and Europe has been associated with local weather at seasonal scales, such as temperature and precipitation, and large-scale climatic drivers at inter-annual and inter-decadal scales, including the solar sunspot cycle, North Atlantic Oscillation (NAO), and El Niño Southern Oscillation (ENSO)^8^,^9^,^10^,^11^,^12^. In addition, anthropogenic influences at both the global and local scales may be contributing to changing ice phenology through alterations in climate, human development, and land-use change^4^. Most past work has been unable to extend statistical analyses of direct observations of climate change and variability any earlier than the 1830’s^4^,^13^. Our paper extends analyses of ice seasonality based on direct human observations of annual ice freeze and breakup to the past 320–570 years. Two inland waters have such long periods of observation – Lake Suwa, Japan, with ice freeze dates from 1443–2014 and Torne River, Finland, with ice breakup dates from 1693–2013.

The Suwa and Torne ice records were collected primarily for religious or economic purposes. The long-term ice freeze record for Lake Suwa, defined as the complete freezing of the lake surface, was collected by Shinto priests observing a legend whereby the male god Takeminakata would cross the lake to visit the female god Yasakatome at her shrine on the other side of the lake. The crossing was evidenced by the god’s footsteps on the ice that left a sinusoidal ice ridge known as the *omiwatari* in Japanese. This important event was followed by a purification process by the priests at the shrine and a celebration that was recorded by at least 15 generations of Shinto priests since 1443. The starting point and direction of the ridge was used to forecast the harvest, temperature, and precipitation for that year^14^,^15^,^16^.

The record for the date of ice breakup for Torne River, defined as when the ice was generally moving, was started by a merchant named Olof Ahlbom in 1693. The timing of ice breakup continued even after Olof Ahlbom fled the Russian troops and then returned to Tornio, Finland following the end of the Russian occupation from 1715–1721. The ice breakup time series has been preserved because of Torne’s important role in trade, transportation, food, and recreation beginning in the 17th and 18th centuries. Logistical factors include: i) proximity to two towns, Tornio, Finland and Haparanda, Sweden with their own newspaper and monitoring organizations, ii) weather journals conserved by Anders Hellant, and iii) ice breakup guessing competitions for which competitors guess the hour and minute of the annual spectacular ice breakup helped preserve the record^17^,^18^,^19^. Kajander thoroughly documented the ice breakup dates and observations for each year from multiple sources^18^.

The two systems differ greatly in geography and local conditions. Suwa is a shallow temperate lake (36.04 N, 138.08 E) with a mean depth of 4.7 m and area of 13.3 km^2^. The Tenryu River flows through Suwa and the lake’s water volume turnover is every 39 days. Suwa has recently developed hot springs surrounded by four resort towns with a population of 91,000 inhabitants^14^,^15^. Torne is a northern river flowing southward into the Baltic Sea from the Arctic. It is one of the largest unregulated rivers in Scandinavia with a length of 522 kilometers and a watershed basin of 40,145 square kilometres. The record site is at 65.84 N, 24.82 E. Here the river flows through a forested valley and two small towns, Haparanda, Sweden, and Tornio, Finland, populated by 15,000 people^19^,^20^. Torne’s break-up is both a thermal and dynamic process. The drainage basin hydrology is snowmelt dominated, and mid-winter break-ups do not occur. The spring hydrograph is usually twin-peak, with flood peak of lowland forest snowmelt coming first and then later mountain snow melt peak. Ice breaks-up in Tornio when the snow melt from low-lands starts to increase the runoff/discharges and usually by that time the solar radiation has decayed ice structure ‘ready’ for break-up^19^,^20^.

The extent and annual grain of the Suwa and Torne ice records allowed us to address how climate and variability differed before and after the start of the Industrial Revolution. More specifically, after the start of the Industrial Revolution: i) Do ice dates reveal more rapid warming? ii) Do warm extremes increase in frequency? iii) Does inter-annual variability increase? iv) Do frequencies of quasi-periodic dynamics (i.e., oscillations of periods with different lengths) change? and v) Do drivers (i.e., explanatory factors) of ice seasonality change?

## Results

### Trends in ice seasonality

**Figure 1:**
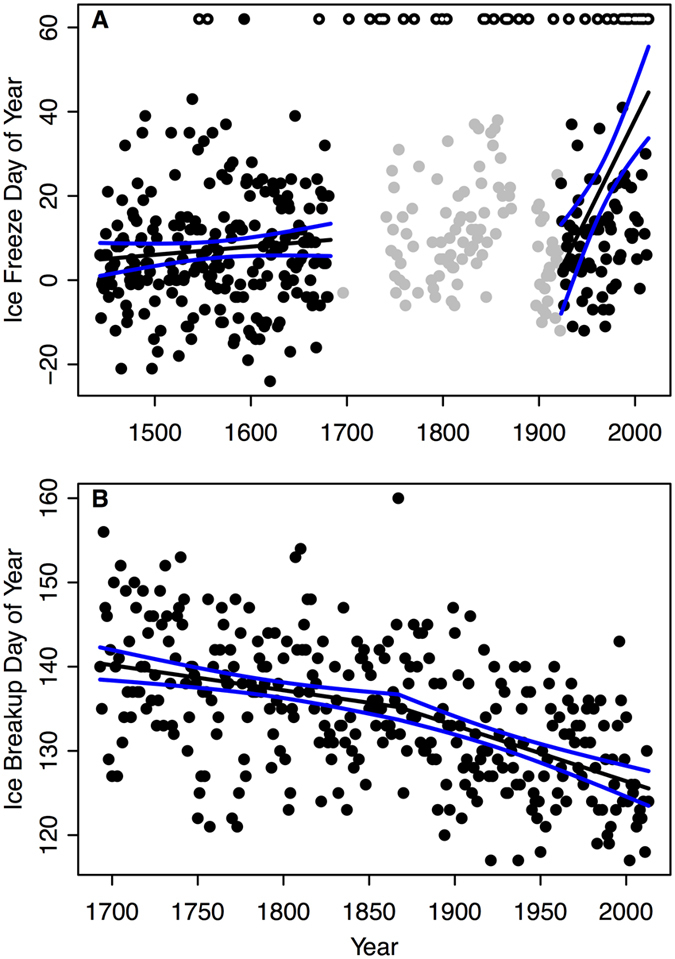
Long-term observations of ice dates for Lake Suwa in Japan. (**A**, ice freeze) and for the Torne River in Finland (**B**, ice breakup). Black dots indicate ice dates. Solid black lines indicate the best fit line of mean ice dates, and solid blue lines bound the 95% confidence interval around the estimated means. For Suwa (**A**), black dots with white centers indicate years when the lake did not freeze and gray points indicate years where the ice dates were considered unreliable (please see Methods for details).

Both Suwa and Torne exhibited more rapid rates of change consistent with warming with later ice freeze and earlier ice breakup following the start of the Industrial Revolution (Fig. 1). For Suwa, the trend in the freeze date in the earlier time period (1443–1683) was 0.19 days per decade and increased to 4.6 days per decade in the more recent time period (1923–2014; Fig. 1A). For Torne, a segmented regression defined two time periods with different slopes and a breakpoint (shift in slope) in 1867. The trend in ice breakup date for Torne decreased from −0.30 days per decade before the breakpoint (1693–1866) to −0.66 days per decade after the breakpoint (1867–2013; more negative values indicate earlier ice breakup; Fig. 1B).

### Extreme events

**Figure 2:**
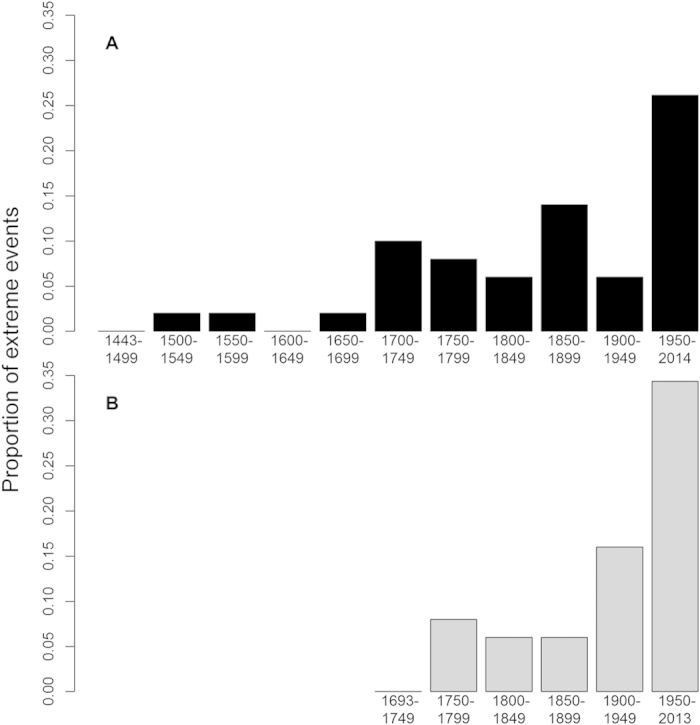
Proportion of extreme warm events over time as indicated by ice dates. In Suwa (**A**, black), a year was extreme if the lake did not freeze. In Torne (**B,** gray), a year was defined as extreme if ice breakup date was before day of year 124 (see Methods for rationale). Note: the first and most recent period included the few earlier or later years.

The prevalence of extreme warm years has been increasing over time for both Suwa and Torne (Fig. 2). Suwa did not freeze five times in the past 10 years (2005–2014) and twelve times in the most recent 55-year period (1950–2004) compared with only three times for a 255-year period from 1443–1700 (Fig. 2A). For Torne, we defined extreme warm years as ice breakup dates prior to day 124 (early May), which corresponds to the top 10% of warmest years. Torne experienced 9 extreme warm years in the 14-year period between 2000–2013 and 10 extreme warm years in the 207-year period between 1693-1899 (Fig. 2B).

### Variability

**Figure 3:**
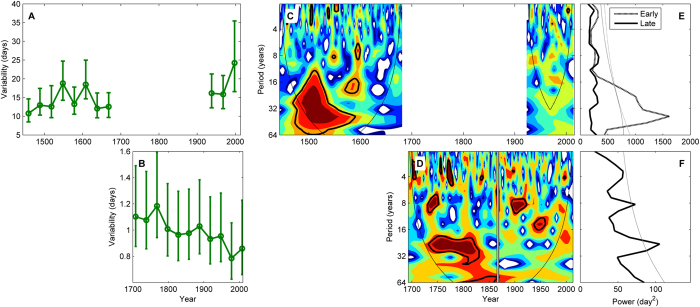
Ice date standard deviation with 95% confidence intervals using a 30-year window for Lake Suwa **(A)** and Torne River (**B**). Visualization of a continuous wavelet transform using a Morlet wavelet for Suwa (**C**) and Torne (**D**) across the full time series, with warm colors indicating high spectral power and cool colors low power, and with vertical lines indicating the breakpoint as identified in Fig. 1. The time-averaged global wavelet transform showing power distribution across frequency (black lines) with a the 95% level of significance (thin grey lines) over the entire records for early and late periods of Lake Suwa (**E**) and Torne River (**F**).

For Suwa and Torne, the timing of ice-freeze and breakup indicate that interannual variability differs among some 30-year non-overlapping windows (Fig. 3). For Suwa, there was no trend in interannual variability in the early period. In the most recent years, increasing extreme no freeze years may be contributing to the increasing inter-annual variability (Fig. 3A). In Torne, variability appears to be decreasing from 1700 to 2013 (Fig. 3B).

### Quasi-periodic dynamics

Before the Industrial Revolution, the ice records of Suwa and Torne both exhibited inter-annual and multi-decadal quasi-cycles with significant periods between 8 and 64 years (Fig. 3C–F). Torne also revealed significant inter-annual quasi-cycles with shorter periods of 2 to 8 years. Following the start of the Industrial Revolution, there were no significant quasi-cycles at any periods for the Suwa ice record, while for Torne only quasi-cycles with shorter periods in the range of 4–16 years persisted. The longer periods present at the beginning of the ice record and before the start of the Industrial Revolution were not apparent in the recent time period for either Suwa or Torne (Fig. 3C,D).

### Important Drivers

**Figure 4:**
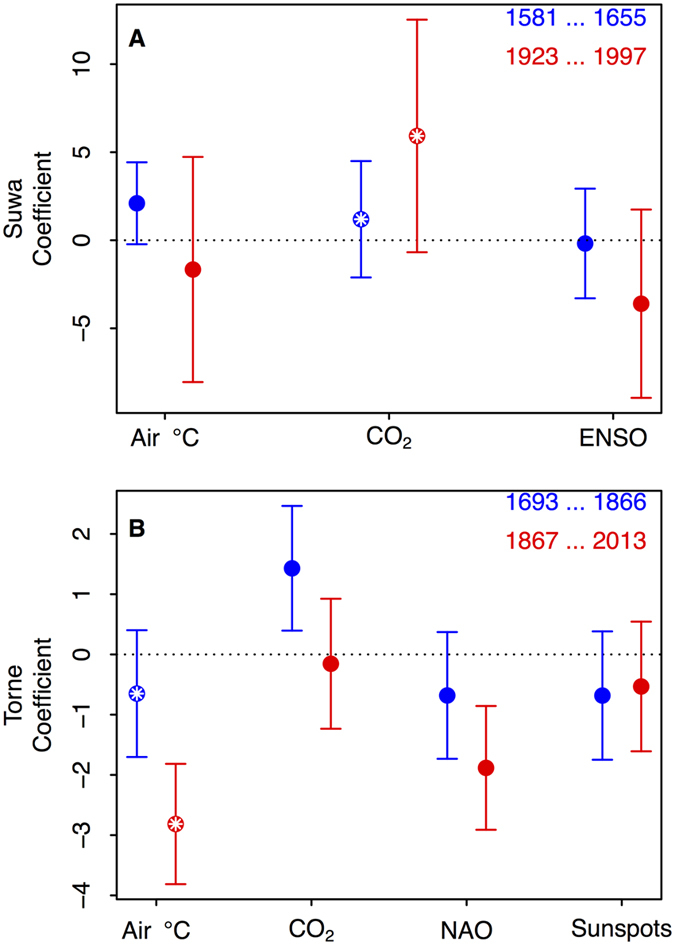
Standardized, detrended regression coefficients relating ice dates to potential drivers for Lake Suwa (**A**) and Torne River (**B**). The potential drivers included: air temperature (Air °C), atmospheric CO_2_, El Niño Southern Oscillation index (ENSO, Suwa only), North Atlantic Oscillation index (NAO, Torne only), and Sunspots (Torne only). Points indicate estimated slope coefficients, with blue being the slope estimated from data in the early time period, and red the more recent time period. Error bars are the 95% confidence intervals. Pairs of colored dots inlaid with white asterisks indicate significant (α = 0.05; maintained within each system) difference between slopes in the early and recent time periods. For Suwa, positive slopes suggest that the potential driver is related to later ice freeze date. Conversely for Torne, negative slopes indicate that the potential driver is related to earlier ice breakup.

The most important explanatory factors for ice freeze and breakup dates were atmospheric carbon dioxide (CO_2_) concentrations and local seasonal air temperatures (March for Suwa and January-April for Torne). In addition, NAO was significant for Torne after the start of the Industrial Revolution, while for Suwa ENSO was not a significant predictor in either period (Fig. 4). Importantly, the significant relationships between ice dates and atmospheric CO_2_ concentrations for Suwa, and ice dates and air temperatures for Torne were apparent only after the onset of the Industrial Revolution. For air temperatures, effect of warmer winters on delaying the Suwa ice date was significantly greater than the effect before the start of the Industrial Revolution (Fig. 4A), and warmer springs were correlated with earlier ice breakup dates in Torne after the start of the Industrial Revolution (Fig. 4B).

## Discussion

Both Suwa and Torne, two inland water bodies from geographically distant areas, reveal increasing trends after the start of the Industrial Revolution consistent with warming with later ice freeze or earlier ice breakup. In both waters, increased rates of warming begin to occur near the start of the Industrial Revolution. In recent years (1923–2014), ice freeze in Suwa is 4.6 days per decade later or approximately 24 times more rapid than the early period (1443–1683), whereas ice breakup in Torne is 0.66 days per decade earlier or twice as fast than the early period. For Suwa, additional evidence of increased rates of warming starting in the 1810’s is provided by diaries of temperature and precipitation^14^,^21^, Lake Nakastuna paleo-reconstructions of sediment cores^16^,^22^, and cherry blossom flowering times in Japan^23^,^24^. For the Torne ice record, 1867 was the coldest spring since 1756 (April-May temperatures) in both Haparanda and Stockholm, Sweden^19^. For three Finnish lakes, 1867 was also the year with the latest ice breakup on record^25^. The Torne breakpoint coincides with the regional end of the Little Ice Age, warming air temperatures, and increasing human population consistent with weather and paleo-records^19^,^20^.

Although these two inland waters have uniquely long time series including years before the Industrial Revolution, there are hundreds of inland waters exhibiting patterns of later ice freeze and earlier ice breakup after the start of the Industrial Revolution^4^,^5^,^6^,^7^,^11^,^13^,^26^,^27^. For example, for 39 records on lakes and rivers across the Northern Hemisphere between 1846 and 1995,the freeze dates were 0.57 days per decade later and 0.63 days earlier per decade^4^. Rates of change in freeze and breakup date did not differ statistically between lakes and rivers^4^. The trends for breakup in Torne were similar, however Suwa differed because the Suwa data in the early 1800s was not used because of a changing and inconsistent Japanese calendar^4^. Subsequent analyses across lakes in the Northern Hemisphere between 1855–2005, warming rates for freeze averaged 1.08 days per decade later for 9 lakes and 0.89 days per decade earlier for breakup for 17 lakes^5^, and as high as 3.7 days per decade for Toronto Harbour, Canada^4^. In the most recent 30-year period, rates of change in ice seasonality were faster such that, ice freeze averaged 1.59 days per decade later for 38 lakes and ice breakup was 1.87 days per decade earlier on average for 66 lakes^5^ and as high as 4.7 days per decade for lakes in New England^13^ and 7.25 days per decade for 402 small, shallow lakes near Barrow, Alaska^27^. Rivers across the Northern Hemisphere have experienced similar trends in earlier ice breakup, later ice freeze or shorter ice duration^6^, including Nemunas River, Lithuania (1 day per decade earlier breakup and freeze)^28^, St. John River, North America (1.1 days per decade earlier breakup and freeze)^29^, and the Yukon River, Canada (0.5 days per decade earlier breakup)^30^. Similarly, direct human observed cherry blossom phenology records in Japan suggest that recent years have been warmer as cherry blossoms have been flowering earlier in recent years than at any point in the past 1200 years consistent with climate warming and urban heat islands in Kyoto, Japan^23^,^24^. These direct human observations coincide with 1209 sets of multiple proxy-based data from tree rings, corals, and ice cores which have illustrated that surface air temperatures globally are warmer in recent years than in the past 1300–1700 years^31^.

The frequency of extreme warm years has been increasing over time both for Suwa and Torne (Fig. 2). For example, in the last decade (2005–2014), Suwa did not freeze five times compared with three times from 1443–1700. Similarly, Torne has experienced 5 extreme warm years in the past decade (2004–2013) compared with 4 extreme warm years between 1693 and 1799. Benson *et al.*^5^ showed that the odds of 10-year, 25-year, and 50-year extreme events have increased for late ice freeze, early ice breakup and shorter ice duration for lakes across the Northern Hemisphere between 1855–2004. Increased prevalence of extreme events in ice seasonality coincide with instrumental, tree-ring, ice-core, and lake sediment records that suggest that the magnitude and frequency of air temperatures extremes at northern latitudes are unique since 1400^32^. The increased prevalence of extreme warm events in ice phenology for Suwa, Torne, and other inland bodies of water across the Northern Hemisphere suggest that a change in mean climatic conditions is contributing to the increase in extreme events^5^,^33^.

Characterizing patterns of interannual variability are complex for Suwa and Torne. Periods of increasing and decreasing variability are apparent. For Suwa, in the early period, there was no trend in interannual variability. The recent period for Suwa shows the highest variability, with the most recent 30 years being the most variable observed across the entire period of record. On the other hand for Torne, the interannual variability in the timing of ice breakup is consistently decreasing. A 90-year record of air temperature^34^ and a 100 and 150-year records of annual ice freeze and breakup dates for Northern Hemisphere lakes^5^ suggest declining climate variability. In contrast, several shorter-term studies on ice freeze and breakup dates across the Northern Hemisphere suggest an increase in variability over time^35^,^36^. Benson *et al.* concluded that the increase in warm extremes resulted primarily from change in the mean^5^. These increases in extremes persisted in Suwa and Torne during periods of increasing as well as decreasing variability. Thus the increase in extreme warm years can be attributed to changes in the mean owing to a trend to warmer years, rather than a change in variability. This numerical explanation for increasing extremes in ice dates and thus temperature, should not be taken to mean that this explanation holds for other types of climatic extremes.

Contrary to our expectations, the same quasi-periodic dynamics did not persist throughout the time series for either Suwa or Torne. Rather, quasi-periodic dynamics tended to weaken and we observed a loss of interdecadal periodicities after the start of the Industrial Revolution. Climate studies have suggested changing periodicities in the North Atlantic Oscillation (NAO) and El Niño Southern Oscillation (ENSO) over time^37^,^38^,^39^,^40^,^41^,^42^. For example, an apparent shift in the importance of inter-year cycles of the NAO has been reported^40^, such that increasing CO_2_ concentrations may have stabilized the positive phase of the NAO. The stabilization of the NAO in the positive phase associated with increasing CO_2_ concentrations appears to be contributing to changing ice phenology in the direction of a warming climate^40^. Similarly, the contribution from interdecadal periods in the NAO index was almost absent from 1940 to the 1970s^37^,^38^. Further, an ENSO reconstruction over the past 1100 years using the North American Drought Atlas^41^ suggested a shift in ENSO cycles to shorter periodicities. For example, 30-year cycles were associated with ENSO between 1500 and 1800 shifting to 2–8 year cycles in the contemporary time period^41^. These changes in significant periodicities over time may imply a structural change in teleconnections among large-scale climate drivers in a warming climate.

Following the start of the Industrial Revolution, increasing air temperatures and atmospheric CO_2_ concentrations (also a proxy for radiative forcing) were important explanatory factors for later ice freeze in Suwa and earlier ice breakup in Torne. Similarly, Tanaka and Yoshino documented a correlation of 0.77 between winter air temperatures (December-January) and freeze date in Suwa between 1945–1978 indicating that warmer winters are associated with later ice freeze^43^. In Suwa, for every degree increase in winter temperatures (December to February) results in 20 days less ice cover^44^. There are also strong correlations (r = 0.76) between April and May air temperatures and Torne ice breakup dates^15^ suggesting that ice breakup date is earlier as local spring air temperatures increase^17^,^19^,^20^,^45^,^46^,^47^. Increases in air temperatures have been associated with earlier ice breakup and later ice freeze for lakes in Finland^46^, Wisconsin, USA^10^, south-central Ontario^12^, and around the Northern Hemisphere^5^,^6^,^48^. Moreover, the relationship for ice breakup for 196 Swedish lakes and air temperature appears to be non-linear suggesting that the rates of warming for lakes in colder regions may become even more rapid under scenarios of climate change^36^. Additional climatic factors such as precipitation, cloud cover, incoming solar radiation, and wind events may also be influencing the timing of ice freeze and breakup^9^,^10^,^45^.

Suwa is influenced by several anthropogenic factors including: land use, human population, flood control gate, and hot springs. The Suwa watershed is 40 times larger than the lake and includes 50% forest and wilderness, 11% rice paddies and dry fields, 6% residential, and the remaining land use includes forest preserves, golf courses, roads and rail corridors, and an amusement park^49^. Four relatively small communities are situated around the shoreline with a combined population of 98,000^50^, small by comparison with the Kyoto metropolitan area with 2,583,304 persons where an urban heat island is important^23^. A low flood control gate is situated at the lake outlet as flooding of the shoreline has been an issue for hundreds of years. Water levels are lowered in the flood season from June 1 to October 15 to receive inflows from 31 inlet streams. Flows at the water gate in September 2015 ranged from at least 49 to 97 m^3^ sec^−^1 with a maximum capacity of 600 m^3^ sec^−1^ 51. Recorded history of Suwa’s hot springs first appeared in Kamakura period (1185–1333). In 1945, when the land was raised to encompass a hot spring near the shoreline, a geyser was developed that initially shot 40 or 50 m into the air. However, presently the geyser is essentially nonexistent and compressed air is used to shoot a small geyser 5 m into the air for viewing by tourists^52^. Relatedly, pumping of hot water from the Kamisuwa-Onsen (hot spring) has increased from 6,000 m^3^ day^−1^ in 1926 to 11,000 m^3^ day^−1^ in 1959 to 15,000 m^3^ day^−1^ in 2015^53^. In the city of Suwa alone, there are wells in seven locations that pump out about 15,000 m^3^ day^−1^ to approximately 13,000 households, at an average temperature of 65 degrees celsius. Evidently, the waters from the hot spring is now almost all being used for human consumption. Although Suwa is influenced by a variety of local factors, the urban heat island, hot springs, and geyser do not provide substantial heat input to the lake to explain the recent prevalence of no-freeze years and later freeze dates alone. Even with these local changes, the ice thickness and ice duration have been strongly related to air temperatures presenting a strong signal of climate change and variability^43^,^44^. Climate warming appears to be the predominant factor in explaining recent rapid warming of Suwa ice dates^15^,^44^.

Torne is one of the largest unregulated rivers in Scandinavia, but is influenced by several local anthropogenic factors including urban development, bridge construction, hydropower development on a tributary of Torne, and demolition of constructed dams^19^,^20^,^45^. For example, the population of central Tornio has increased from 1000 inhabitants in 1880 to 10,000 in 2009^19^. In addition, several bridges were constructed including the railway bridge in 1910 and a highway bridge in the 1930s and 1970s^17^. Three small hydroelectric power stations have been developed on one of Torne’s tributary rivers (Tengelionjoki) in 1954 (2.5 MW), 1955 (0.5 MW), and 1987 (10.5 MW)^19^. In the 20^th^ century, 162 log-driving dams were constructed on Torne, but later demolished in the 1970s that may have sped up the runoff^17^,^20^,^45^. Despite the number of anthropogenic factors on Torne, the human influence on Torne is small relative to the size of the river and no statistically significant consequences of local anthropogenic activities on ice breakup dates in Torne are apparent^19^.

These analyses based on direct human observations of ice for Suwa and Torne over 570 and 320 years are consistent with climate change based on later ice formation and earlier spring melt, without relying on instruments, modelling results, or inferences from paleo records. We advance knowledge of climate change and variability using two rare datasets that are well within the direct human perception of the world. As Suwa no longer freezes every year, the male god, Takeminakata, is no longer able to walk across the lake to see the female god, Yasakatome, every year^15^. If atmospheric CO_2_ emissions and air temperatures continue to rise, the male god may soon never cross the lake again to visit the female god as he has in Shinto legend for centuries.

## Methods

### Ice freeze and breakup dates

We obtained ice freeze dates for a 572-year period from 1443–2014 for Lake Suwa and a 321-year record of river ice breakup dates for Torne River from 1693–2013 from the National Snow and Ice Data Center^54^,^55^. Leap years have been taken into account for both Suwa and Torne.

Ice-freeze dates for Suwa were first recorded in 1443. Ice freeze date, defined as the first date of complete cover, was decided by observers from the shoreline. The name of the family observing ice freeze is provided, although the unique name of the observer is not given but would have included at least 15 generations of observers^16^. The Shinto Shrine also reported the Omiwatari date of ice ridge formation. Fujiwhara^14^ wrote that of the various ice phenomena recorded by the Shinto Shrine, first complete ice cover was the most robust^14^,^16^. Unfortunately, there are missing data in the midyears of the time series (1505–1515) and more importantly, data from 1682–83 to 1922–23 are considered unreliable for analysis ice cover freeze dates^14^,^16^,^43^,^56^. In those middle years, various changes in the calendar confused the record, ice cover dates often were indicated as approximate or were not provided even though the lake did freeze over, the group making the observations varied, and Omiwatari date or even the Omiwartari ceremony often were substituted for the ice cover date. We eliminated all data from 1682–1923 from the analyses to reduce the uncertainty in dates of ice freeze^14^,^16^,^43^,^56^. However, the ice-freeze date between 1443–1682 and 1924–2014, in addition to the presence or absence of lake freeze from 1443–2014 are considered to be very reliable^14^,^16^,^43^,^56^. For years when more than one data source was available (1897–1993), we numerically compared the values. In almost all years they were the same and if not, the standard deviation between the values between 1944 and 1996 was 2.65^16^. When they were not the same, we used Arakawa^14^ over the Suwa Meteorological Observatory and Yatsurugi Shrine from 1443 to 1953; from 1953 to 1993 we used the Suwa Meteorological Observatory over the Yatsurugi Shrine, and from 1994 to 2014 we used Yatsurugi Shrine. There were 3 exceptions to these choices (1950 we used the Suwa Meteorological Observatory; 1952 and 1976 where we used information from Tadashi Arai; Supplementary Table 1). Ice freeze dates occur before and after January 1^st^, therefore we converted dates to day of year, using a zero to represent the calendar day January 1^st^.

Ice breakup dates (defined as the general movement of ice) from Torne have been recorded since 1693 and were also converted to day of year. Data were collected from observation journals, newspapers, weather monitoring services in Tornio, Finland, and Haparanda, Sweden, the Finnish Environment Institute, and local guessing competitions^17^,^18^ (Supplementary Table 2). Ice breakup data appear to be homogeneous even in the presence of different observers as many years have multiple observers, although the margin of error may be approximately 2.1 days^18^,^19^,^20^. For complete details of the record, please see Kajander, which documents and compares every ice breakup date from a variety of sources, in addition to providing a translation of the original comments provided by the observer^18^.

### Large-scale climate drivers and weather

We obtained data from paleo- and historical records that may be important to ice freeze date on Lake Suwa and ice breakup date on Torne River (Supplementary Data Table 1). We acquired average annual sunspot number from 1700–2012 from the Solar Influences Data Analysis Center^57^. The average annual sunspot number represents a relative index of solar activity for the visible solar surface based on visual observations from a group of people from different regions around the globe^57^. Atmospheric carbon dioxide concentrations from 1AD -2012 AD were acquired from proxies of Antarctic ice cores from 1AD - 1957 AD and direct observations from 1958–2012 averaged from Mauna Loa, Hawaii and the South Pole from the Scripps CO_2_ program^58^. We acquired an index of El Niño Southern Oscillation (ENSO) from 1301–2005 derived from the North American Drought Atlas. Thousands of tree-ring records from North America were used to produce an annual database of drought reconstructions and subsequently analyzed using Empirical Orthogonal Functions (EOF) to develop an annual ENSO index^59^. In addition, we acquired a winter (December-March) North Atlantic Oscillation Index based on a combination of reconstruction and instrumental data from 1659–2013. Between 1659 and 1864, reconstructions of temperature, precipitation, and other proxy data were used based on observations of ice, snow, and tree-ring data^60^. From 1864–2013, instrumental data were used to develop the NAO index which has been defined as the pressure difference from stations in Ponta Delgada, Azores and Reykjavik, Iceland^61^.

We acquired air temperatures for both locations. For Suwa, we obtained reconstructed March air temperatures from Kyoto, Japan that were derived from cherry blossom phenology records obtained from diaries of emperors, aristocrats, and monks from 854–1995^62^. The DTS model (DTS: the number of days transformed to standard temperature) was based on cherry blossoming data and used to develop a March mean temperature reconstruction model. An exponential model was developed for March air temperatures using rate of plant development^62^. For Torne, we acquired reconstructed January to April air temperature data from Stockholm, Sweden for 1500 to 2008^63^. Ledgers documenting the fees and taxes, in addition to diaries and records related to harbour activities in the Stockholm harbour, were used to identify the start of the sailing season each year from which a winter/spring air temperatures were reconstructed^63^. Leijonhufvud *et al.* compare, calibrate, and evaluate over 15 sources of data summarizing sailing records in the Stockholm harbour in an effort to validate the reconstruction of January-April air temperatures^63^.

### Trends in ice seasonality

#### Continuous Segmented Regression

We used segmented regression to test for abrupt changes in the trend of ice dates in Torne. Specifically, we wanted to test when a shift in the temporal trend of ice date may have occurred. To estimate the timing and magnitude of a change in the slope of ice dates, we used continuous segmented regression (CSR) models. In CSR, trend lines on either side of the estimated breakpoint intersect (hence making them “continuous”), but are allowed to have different slopes. In general, a CSR takes the form


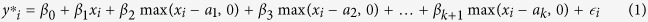



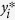
 is a latent variable representing ice dates, x_i_ are the years of the time series, β_0_ is the intercept of the regression (ice date on year 0), β_1_ is the trend in ice date prior to any breakpoint (ice date per year), the a_k_ are the breakpoints (k was either 1 or 2 for this study. Because the number of parameters increases with k, we limited k to avoid over-fitting the model), the β_k+1_ are the changes in the temporal trend at each of the k breakpoints compared to the trend prior to the breakpoint, and the ε_i_ are the errors. Note that the β_k+1_ parameters indicate the effect on ice date of years elapsed since the previous breakpoint once the breakpoint has passed.

#### Fitting CSR in Torne (Ordinary Least Squares (OLS) Regression)

The Torne time series began in 1693 and ended in 2013, thus x = 1, 2, … 321. Ice breakup dates for Torne ranged from day 117 to day 160, and the ice melted each year of the time series. For Torne, we fit CSR parameters using ordinary least squares using the lm() function in the statistical programming language R.

#### Fitting Regressions in Suwa (Tobit)

The Suwa time series was split into an early period (1443–1683) and a late period (1923–2014) (Fig. 1). A breakpoint model was not fit for Suwa; rather, separate linear regressions were fit for the early and late periods of the time series, with the parameters being the intercept and the effect of years elapsed on ice date. The day that Suwa froze ranged from day -24 to day 62 (negative values indicate freezing before January 1^st^ of the designated “year”); however, there were 37 years when the lake did not freeze. Treating no-freeze years as missing data or as a constant date would result in biased results if we employed the regression techniques used for Torne. Thus, calculating trends for Suwa ice dates required a statistical approach distinct from that used in Torne. We can consider the lake an instrument that measures a value which we call ice date. This instrument indicates the favorability of conditions for ice formation, and if we understand the lake instrument to censor measurements at 62, then the no-freeze years can be encoded as ice dates of 62. We consider Suwa as an instrument with output of ice date that is censored at an upper limit, L = 62. As such, the observed y_i_ are related to L and the latent variable 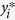 in the following manner:


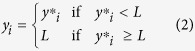


To address this censoring of Suwa ice dates while fitting the parameters, we used a Tobit regression model. For a Tobit regression model with an upper limit (right censoring) of the response variable, the log likelihood of observing data given the parameters β (as in Eq. 1) and σ^2^ (the variance of ε in Eq. 1), can be calculated as:


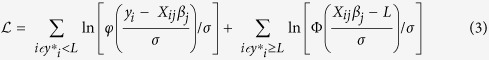


where φ(.) and Φ(.) are the probability and cumulative density functions of the normal distribution, respectively. The first term is the standard normal likelihood, and applies to observations for which an ice date was observed. The second term reflects the probability of the observation being censored, and applies to no-freeze years. Given parameter values, Eq. 3 reflects the probabilities of observing the ice dates (y_i_) during freeze years, as well as the probabilities that ice date was censored (unobserved) during no-freeze years. Thus, the β in the Tobit regression model indicate the effect of unit change in X on the latent variable, 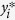. We used Tobit regression models as implemented by the vglm() function in the R package VGAM to fit parameters to Suwa data.

#### Finding Breakpoint Locations

When fitting models with one breakpoint, breakpoints were searched exhaustively, and the breakpoint location whose model had the lowest Akaike Information Criterion (AIC) was selected. The model with the lowest AIC value was chosen as the most parsimonious model^64^. The same procedure applied to fitting the two-breakpoint model for Torne, which was fit with OLS (Supplementary Fig. 1).

#### Model Selection

We compared AIC values from CSR models containing one or two breakpoints to multiple regression models containing only year or only year and year^2^ as predictor variables. AIC is calculated from the negative log likelihood of the data given the model, minus a penalty of 2 AIC points per parameter; lower AIC values indicate better model fit. A model with only year as a predictor has two regression coefficients (β’s for intercept and linear slope), both the polynomial and the single breakpoint model have three, and the two breakpoint model has four.

Like the breakpoint models, the polynomial model indicates that trends in ice date are becoming steeper over time. The four models were of similar AIC for the Torne data, with the AIC decreasing as model complexity increased from 2155.8 to 2152.8 (Table S3). Comparing AIC values among models indicates that a single linear trend in ice date is a poorer description of the data than the more complex models that indicate accelerating ice dates, and that the single breakpoint model fits the data about as well as or better than the other models.

#### Inter-annual variability

For gross climate variability, we analyzed the standard deviation of non-overlapping 30-year windows of ice dates. Although all permutations of window sizes were evaluated, the general pattern was not influenced by window size. Therefore, we chose 30-year windows to assess long-term variability in climate by accounting for intra-annual, inter-annual and most multi-decadal variation attributed to weather, Quasi-biennial oscillation, El Nino Southern Oscillation, solar sunspots, and shorter multi-decadal variation in the range of 20–30 years. Before the standard deviation was calculated, any linear trend in the data within the 30-year window was removed to remove potential bias due to trend. For Suwa, the missing (unobserved) ice dates were removed, reducing the number of observations in the window and widened the standard deviation confidence interval. No-ice years were treated as censored values and included in the maximum likelihood fit of standard deviation (Matlab 2011a *normfit* function). All possible 30-year non-overlapping windows were examined to ensure the pattern in variability was not sensitive to the specific start-date used. The variability is presented in units of *days* and differs in baseline magnitude between the two lakes.

#### Quasi-periodic dynamics

To examine the quasi-periodic dynamics in the ice data through time, we applied a continuous wavelet transform to the ice dates for both lakes. This allowed for the decomposition of the signal into its individual frequency components while still examining how they change through time (as opposed to Fourier Transform). For the Suwa data wavelet analysis, the omitted middle period contained the majority of missing values. The missing values in the early and late period (∼10% of values) were replaced with the average of the time series, a conservative approach to prevent spurious oscillation detection that can occur with interpolated datasets. Tornio had no missing or no-ice observations. The Morlet basis function was chosen for its frequency identifying characteristics. For the continuous wavelet transform (Fig. 3C,D), significant periods were identified using a chi-squared test with 95% confidence intervals and assuming a red noise mean background spectrum. For the global wavelet transform (Fig. 3E,F), a 95% significance was also calculated using a red noise background spectrum, but time-averaged across all times outside of the cone of influence to give an overall significance level. For detail on the methods used in the continuous and global wavelet analysis, see Torrence and Compo^65^.

#### Changing drivers

We explored linear relationships between ice date and the following climate drivers (unless otherwise specified, drivers apply to both systems): air temperature (Air °C), atmospheric CO_2_, El Niño Southern Oscillation index (Suwa only), North Atlantic Oscillation index (Torne only), and Sunspots (Torne only). In each system, the relationships between ice date and each of the climate drivers were explored using data before and after the breakpoint. In Suwa, the duration of the period on both sides of the breakpoint was 75 years (1581–1655 and 1923–1997), and the time periods were chosen to maximize duration on either side of the breakpoint while minimizing the inclusion of years for which ice data were missing. In Torne, we used the full time series on either side, giving 174 years in the first portion (1693–1866) and 146 years in the second portion (1867–2013).

For each ice date–driver pair in each system, we performed separate linear regressions for an early and late period. In these analyses, we detrended the response and driver variables (i.e., took the residuals from a linear temporal trend), and for the driver variables, we scaled (x_scaled _= (x−μ)/σ, where μ is the mean of x, and σ is its standard deviation) the time series. These transformations were applied separately to early and late periods (Supplementary Fig. 2–8). This procedure resulted in twenty-two separate regressions, and each before-after pair of regressions is equivalent to fitting a single model of the form


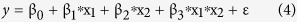


where y is ice date, β_0_ is the intercept, x_1_ is the driver variable and β_1_ its effect, x_2_ is a dummy variable that is 0 before the breakpoint and 1 after, ergo β_2_ is the post-breakpoint change in the intercept, and β_3_ is the change in the relationship between the driver and ice date after the breakpoint (i.e., β_3_ is the adjustment made to β_1_ after the breakpoint). As described for the analysis of temporal trends, for Torne Eq. 4 was fit with ordinary least squares, and with the Tobit regression for Suwa. For each regression, we assumed there was a possibility that the residuals of the regression would be autocorrelated; to estimate coefficient standard errors in the presence of autocorrelation, we used a bootstrapping procedure where the randomized residuals retained the autocorrelation structure of the regression residuals.

To characterize the autocorrelation structure of the residuals in Eq. 4, we fit an autoregressive moving average (ARMA(p,q)) model with *p* AR parameters and *q* MA parameters. An ARMA(p,q) model has the general form


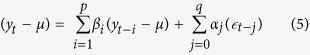


The y_t_ are the residuals ε from Eq. 4, and μ is the mean of the residuals, which is 0. The β_i_ are the AR parameters, the α_j_ are the MA parameters, and the ε_t-j_ are the residuals. We applied Eq. 5 to the ε of Eq. 4. We selected among ARMA models using AIC, and allowed model complexity to vary from ARMA(1, 0) to ARMA(5, 5) (including all orders in between). These models were fit and selected using the stepwise procedure implemented in the auto.arima function in the forecast R package. We then simulated an ARMA process using the fitted ARMA parameters; the variance of the innovations in the simulated ARMA process was the maximum likelihood estimate acquired in fitting Eq. 5 to the residuals.

The ARMA-simulated residuals formed the basis of our bootstrapping procedure. These simulated residuals were then added to the fitted regression values, and the regression was re-fit. This procedure was repeated 1,000 times. The standard deviation of these 1,000 parameter estimates was then used as the standard error of the parameters in Eq. 4. In summary, our bootstrapping procedure was as follows:

1. Fit Eq. 4
2. Separate residuals from Step 1 into before and after period
3. Fit and select an ARMA time series model (Eq. 5) to each set of residuals
4. Use the model from Step 3 to simulate new sets of residuals from fitted time series model
5. Add the residuals simulated in Step 4 to fitted values (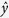 ) from Eq. 4
6. Re-fit Eq. 4 to the new set of observations from the sum in Step 5
7. Repeat Steps 2–6 1,000 times
8. The bootstrapped estimate of the standard error of parameters in Eq. 4 is the standard deviation of the 1,000 estimates from Step 6

We calculated p-values corresponding to the probability that regression coefficients between drivers and ice dates differed before (subscript 1) and after (subscript 2) the breakpoint in the following manner:


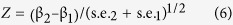


where Z is the z-score to be compared under the standard normal curve, the β are the regression coefficients before (β_1_) and after (β_2_) the breakpoints, and s.e. are the standard errors of those coefficients. We also corrected these p-values to control for multiple tests and to maintain constant family wise error rates. We performed the Holm-Bonferroni correction, and no conclusions about significance among pairs changed.

## Additional Information

**How to cite this article**: Sharma, S. *et al.* Direct observations of ice seasonality reveal changes in climate over the past 320–570 years. *Sci. Rep.* **6**, 25061; doi: 10.1038/srep25061 (2016).

## Supplementary Material

Supplementary Information

## References

[b1] Jones P. D., Briffa K. R., Barnett T. P. & Tett S. F. B. High-resolution palaeoclimatic records for the last millennium: interpretation, integration and comparison with General Circulation Model control-run temperatures. The Holocene 8, 455–471 (1998).

[b2] Mann M. E., Bradley R. S. & Hughes M. K. Global-scale temperature patterns and climate forcing over the past six centuries. Nature 392, 779–787 (1998).

[b3] Masson-Delmotte V. M. *et al.* Information from Paleoclimate Archives. Clim. Chang. 2013 Phys. Sci. Basis. Contrib. Work. Gr. I to Fifth Assess. Rep. Intergov. Panel Clim. Chang. 383–464, 10.1017/CBO9781107415324.013 (2013).

[b4] Magnuson J. J. *et al.* Historical Trends in Lake and River Ice Cover in the Northern Hemisphere. Science *(80-.)*. 289, 1743–1746 (2000). 10976066 10.1126/science.289.5485.1743

[b5] Benson B. J. *et al.* Extreme events, trends, and variability in Northern Hemisphere lake-ice phenology (1855–2005). Clim. Change 112, 299–323 (2012).

[b6] Beltaos S. & Prowse T. River-ice hydrology in a shrinking cryosphere. Hydrol Process 23, 122–144 (2009).

[b7] Prowse T. *et al.* Past and future changes in arctic lake and river ice. Ambio 40, 53–62 (2011).

[b8] Livingstone D. A. Large-scale climatic forcing detected in historical observations of lake ice break-up. Verh Internat Verein Limnol 27, 2775–2783 (2000).

[b9] Ghanbari R. N. *et al.* Coherence between lake ice cover, local climate and teleconnections. J Hydrol 374, 282–293 (2009).

[b10] Sharma S., Magnuson J. J., Mendoza G. & Carpenter S. R. Influences of local weather, large-scale climatic drivers, and the ca. 11 year solar cycle on lake ice breakup dates; 1905–2004. Clim. Change 118, 857–870 (2013).

[b11] Sharma S. & Magnuson J. J. Oscillatory dynamics do not mask linear trends in the timing of ice breakup for Northern Hemisphere lakes from 1855 to 2004. Clim. Change 124, 835–847 (2014).

[b12] Fu J. & Yao H. Trends of ice breakup date in south-central Ontario. J Geophys Res-Atmo 10.1002/2015JD023370 (2015).

[b13] Hodgkins G. A. The importance of record length in estimating the magnitude of climatic changes: an example using 175 years of lake ice-out dates in New England. Clim. Change 119, 705–718 (2014).

[b14] Arakawa H. Fujiwhara on five centuries of freezing dates of Lake Suwa in the Central Japan. Arch. fur Meteorol. Geophys. und Bioklimatologie Ser. B 6, 152–166 (1954).

[b15] Hanazato T. Gods are sad: water quality and the reason for the change in lake water. Science Journal Kagaku 71, 92–99 (2001).

[b16] Ishiguro N. Homogeneity of the Omiwatari records of Lake Suwa as the database for winter temperature estimation. Geographical Review of Japan 74A, 415–423 (2001).

[b17] Kajander J. Methodological aspects on river cryophenology exemplified by a tricentennial break-up time series from Tornio. Geophysica 29, 73–95 (1993).

[b18] Kajander J. Cryophenological records from Tornio. Mimeograph series of the national board of waters and the environment, 189 pp (National Board of Waters and Environment, 1995).

[b19] Loader N. J., Jalkanen R., McCarroll D. & Moberg A. Spring temperature variability in northern Fennoscandia AD 1693–2011. J. Quat. Sci. 26, 566–570 (2011).

[b20] Helama S., Jiang J., Korhonen J., Holopainen J. & Timonen M. Quantifying temporal changes in Tornionjoki river ice breakup dates and spring temperatures in Lapland since 1802. J. Geogr. Sci. 23, 1069–1079 (2013).

[b21] Mikami T. Climatic variations in Japan reconstructed from historical documents. Weather 63, 190–193 (2008).

[b22] Adhikari D. P. & Kumon F. Climatic changes during the past 1300 years as deduced from the sediments of Lake Nakatsuna, Central Japan. Limnology 2, 157–168 (2001).

[b23] Aono Y. & Kazui K. Phenological data series of cherry tree flowering in Kyoto, Japan, and its application to reconstruction of springtime temperatures since the 9th century. Int. J. Climatol. 28, 905–914 (2008).

[b24] Primack R. B., Higuchi H. & Miller-Rushing A. J. The impact of climate change on cherry trees and other species in Japan. Biol. Conserv. 142, 1943–1949 (2009).

[b25] Kuusisto E. An analysis of the longest ice observation series made on Finnish lakes. Aqua Fenn 17, 123–132 (1987).

[b26] Howk F. Changes in Lake Superior ice cover at Bayfield, Wisconsin. J. Great Lakes Res 35, 159–162 (2009).

[b27] Surdu C. M., Duguay C. R., Brown L. C. & Fernandez Prieto D. Response of ice cover on shallow lakes of the North Slope of Alaska to contemporary climate conditions (1950–2011): radar remote-sensing and numerical modeling data analysis. Cryosphere 8, 167–180 (2014).

[b28] Stonevicius E., Stankunavicius G. & Kilkus K. Ice regime dynamics in the Nemunas River, Lithuania. Clim Res 36, 17–28 (2008).

[b29] Beltaos S. Effects of climate on mid winter ice jams. Hydrol Process 16, 789–804 (2002).

[b30] Jasek M. J. 1998 break-up and flood on the Yukon River at Dawson – Did El Nïno and climate play a role? in Ice in Surface Waters (ed Shen H. T. ), p761–768 (Balkema, 1998).

[b31] Mann M. E. *et al.* Proxy-based reconstructions of hemispheric and global surface temperature variations over the past two millennia. PNAS 105, 13252–13257. 18765811 10.1073/pnas.0805721105 PMC2527990

[b32] Tingley M. P. & Huybers P. Recent temperature extremes at high northern latitudes unprecedented in the past 600 years. Nature 496, 201–205 (2013). 23579678 10.1038/nature11969

[b33] Collins M. *et al.* Climate change 2013: the physical science basis. Contribution of Working Group I to the Fifth Assessment Report of the Intergovernmental Panel on Climate Change. *Long-term Clim. Chang. Proj. Commitments Irreversibility, Cambridge Univ. Press. Cambridge, UK, New York* (2013).

[b34] Karl T., Knight R. & Plummer N. Trends in high-frequency climate variability in the twentieth century. Nature 377, 217–220 (1995).

[b35] Kratz T., Hayden B., Benson B. & Chang W. Patterns in the interannual variability of lake freeze and thaw dates. Verh. Internat. Verein. Limnol. 27, 2796–2799 (2000).

[b36] Weyhenmeyer G. A. *et al.* Large geographical differences in the sensitivity of ice-covered lakes and rivers in the Northern Hemisphere to temperature changes. Glob. Chang. Biol. 17, 268–275 (2011).

[b37] Hurrell J. W. & Loon H. Van. in Clim. Chang. High Elev. Sites 69–94 10.1023/A (Springer: Netherlands, 1997).

[b38] Higuchi K., Huang J. & Amir S. A Wavelet Characterization of the North Atlantic Oscillation Variation and Its Relationship to the North Atlantic Sea Surface Temperature. Int. J. Climatol. 19, 1119–1129 (1999).

[b39] Robertson D., Wynne R. & Chang W. Influence of El Niño on lake and river ice cover in the Northern Hemisphere from 1900 to 1995. Int Ver Theor Angew Limnol 27, 2784–2788 (2000).

[b40] Yoo J. & D’Odorico P. Trends and fluctuations in the dates of ice break-up of lakes and rivers in Northern Europe: The effect of the North Atlantic Oscillation. J. Hydrol. 268, 100–112 (2002).

[b41] Li J. *et al.* Interdecadal modulation of El Niño amplitude during the past millennium. Nat. Clim. Chang. 1, 114–118 (2011).

[b42] Cohen J. & Barlow M. The NAO, the AO, and global warming: How closely related? J. Climate 18, 4498–4513 (2005).

[b43] Tanaka M. & Yoshino M. M. Re-examination of the climatic changes in central Japan based on freezing dates of Lake Suwa. Weather 37, 252–259 (1982).

[b44] Arai T. & Pu P. A preliminary study on the water temperature and freeze of Lake Suwa in Japan and shallow lakes in eastern China. Jpn. J. Limnol. 48, 225–230 (1987).

[b45] Zachrisson G. 1989. Climate variations and ice conditions in the River Torneälven. The Publications of the Academy of Finland 9, 353–364 (1989).

[b46] Korhonen J. Ice conditions in lakes and rivers in Finland. The Finnish Environment 751, 1–145 (2005).

[b47] Moberg A., Tuomenvirta H. & Nordli Ø. Recent climatic trends. In: Seppälä M. (ed.) Physical Geography of Fennoscandia, 113–133. (Oxford University Press, Oxford, 2005).

[b48] Magnuson J. J., Benson B. J. & Kratz T. K. Patterns of coherent dynamics within and between lake districts at local to intercontinental scales. Boreal Environ. Res. 9, 359–369 (2004).

[b49] Otsuka K., Fujita K., Isono Y. & Mizuochi M. A Japanese experience with stakeholder involvement in water environment conservation: the case of Lake Suwa. (2011) www.ide.go.jp/English/Publish/Download/Jrp/155.html (Date of access: 22/05/2015).

[b50] Affairs Department Planning Division. Changes in population concentration of district Suwa Region. (2013). http://www.city.chino.lg.jp/. (Date of access: 22/05/2015).

[b51] Suwa Construction Office. Kamaaguchi Floodgate. (2015) www.pres.Nagano.lg.jp/suwaken/jimusho/kamaguchi.html (Date of access: 22/05/2015).

[b52] Japan National Tourism Association. Japan. the Official Guide. Suwa Geyser Center. (2015) http://www.jnto.go.jp/eng/location/spot/natuscen/suwalake-geyser-center.html (Date of access: 22/05/2015).

[b53] Yuhara K. & Seno K. in Balneology. Ch. 2, 56–60 (Chijin-Shokan, Tokyo, 1969).

[b54] Benson B. & Magnuson J. Global lake and river ice phenology database. Boulder, Colorado USA: National Snow and Ice Data Center. (2012).

[b55] Benson B. J. *et al.* Regional coherence of climatic and lake thermal variables of four lake districts in the Upper Great Lakes Region of North America. Freshw. Biol. 43, 517–527 (2000).

[b56] Arakawa H. On five centuries of freezing dates of Lake Suwa (36°N, 138°E) in the central Japan. Chigaku Zashi 63, 193–200 (1954).

[b57] Solar Influence Data Centre. Sunspot Number and long-term solar observations. (2015). www.sidc.be/SILSO/ (Date of access: 22/05/2015).

[b58] Keeling C. D. *et al.* Exchanges of atmospheric CO2 and CO2 with the terrestrial biosphere and oceans from 1978 to 2000. Glob. Asp. SIO Ref. Ser. 01–06 (2001).

[b59] Li J. *et al.* Interdecadal modulation of El Niño amplitude during the past millennium. Nat. Clim. Chang. 1, 114–118 (2011).

[b60] Luterbacher J. Extending North Atlantic Oscillation reconstructions back to 1500. Atmos. Sci. Lett. 2, 114–124 (2001).

[b61] Hurrell J. The Climate Data Guide: Hurrell North Atlantic Oscillation (NAO) Index (station-based). (2015). https://climatedataguide.ucar.edu/climate-data/hurrell-north-atlantic-oscillation-nao-index-station-based (Date of access: 22/05/2015).

[b62] Aono Y. & Saito S. Clarifying springtime temperature reconstructions of the medieval period by gap-filling the cherry blossom phonological data series at Kyoto, Japan. Int. J. Biometeorol. 54, 211–219 (2010). 19851790 10.1007/s00484-009-0272-x

[b63] Leijonhufvud L. *et al.* Five centuries of Stockholm winter/spring temperatures reconstructed from documentary evidence and instrumental observations. Clim. Change 101, 109–141 (2010).

[b64] Burnham K. P. & Anderson D. R. in Model Selection and Multimodel Inference: A practical information theoretic approach. Ch. 2, 60-76 (Springer, New York, 2002).

[b65] Torrence C. & Compo G. P. A Practical Guide to Wavelet Analysis. Bull. Am. Meteorol. Soc. 79, 61–78 (1998).

