# Supplementary material for: Direct observations of ice seasonality reveal changes in climate over the past 320–570 years
Source: Sci Rep. 2016 Apr 26;6:25061. doi: 10.1038/srep25061 (PMC4844970; doi:10.1038/srep25061)
Supplement: Supplementary Information [file srep25061-s1.doc]

**Title:** Direct observations of ice seasonality reveal changes in climate over the past 320-570 years

**Authors:** Sapna Sharma^1*¶^, John J. Magnuson^2¶^, Ryan D. Batt ^2,3¶^, Luke A. Winslow ^2,4¶^, Johanna Korhonen^5^ and Yasuyuki Aono^6^

**Affiliations:** ^1^Department of Biology, York University, Toronto, Ontario, Canada, M3P1J3

^2^ Center for Limnology, University of Wisconsin-Madison, Madison, Wisconsin, USA 53706

^3^ Department of Ecology, Evolution, and Natural Resources, Rutgers University, New Brunswick, New Jersey, USA 08901

^4^ Center for Integrated Data Analytics, United States Geological Survey, 8505 Research Way, Middleton, Wisconsin, 53562

^5^ Freshwater Centre, Finnish Environment Institute, Helsinki, Finland, FI-00260

^6^ Graduate School of Life and Environmental Sciences, Osaka Prefecture University, Osaka, Japan 599-8531

¶ Joint first authors

* Corresponding author email: [sharma11@yorku.ca](mailto:sharma11@yorku.ca)

**Supplementary Material**


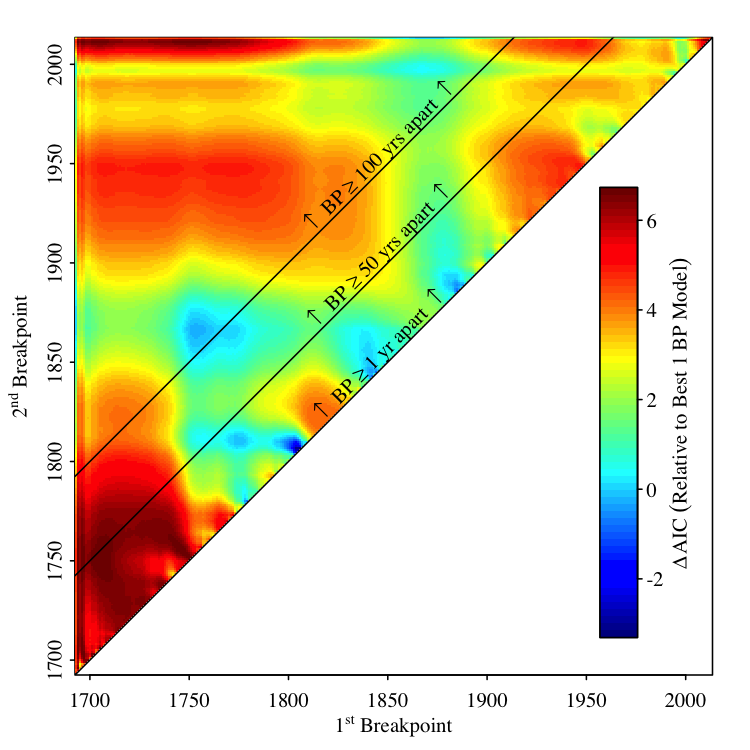


**Supplementary Fig. S1.** Relative probabilities of two versus one breakpoint in the Torne time series. Colors indicate change in AIC for the two breakpoint model relative to the one breakpoint model (Table S3) for all combinations of first and second breakpoint years in the two breakpoint model. Sloped lines indicate boundaries where the first and second breakpoints are separated by the indicated period of time. Note that when at least 25 years separates breakpoints, the one breakpoint model is always more parsimonious than the two breakpoint model (Supplementary Table 3).


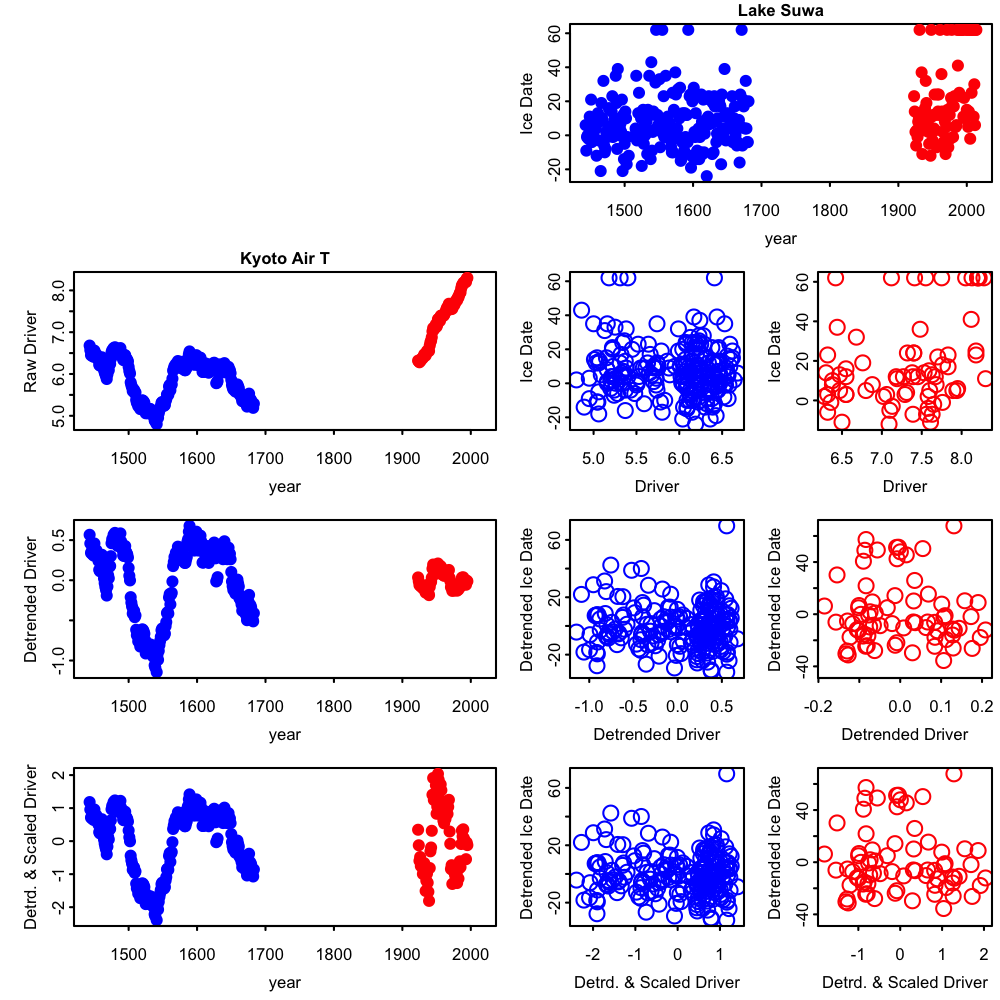


**Supplementary Fig. S2.** Time series of a driver (Kyoto air temperature, ºC) and ice dates (Lake Suwa), and scatter plots of the two (driver on horizontal axis, ice date on vertical axis). Several combinations of driver transformations (untransformed, detrended [residuals of linear regression with years elapsed as the predictor variable], and detrended and scaled [subtract mean, divide by standard deviation]) and time series transformations (untransformed and detrended) are shown. Blue symbols indicate data used for the “early period” analysis in Figure 4, and red symbols the “late period”.


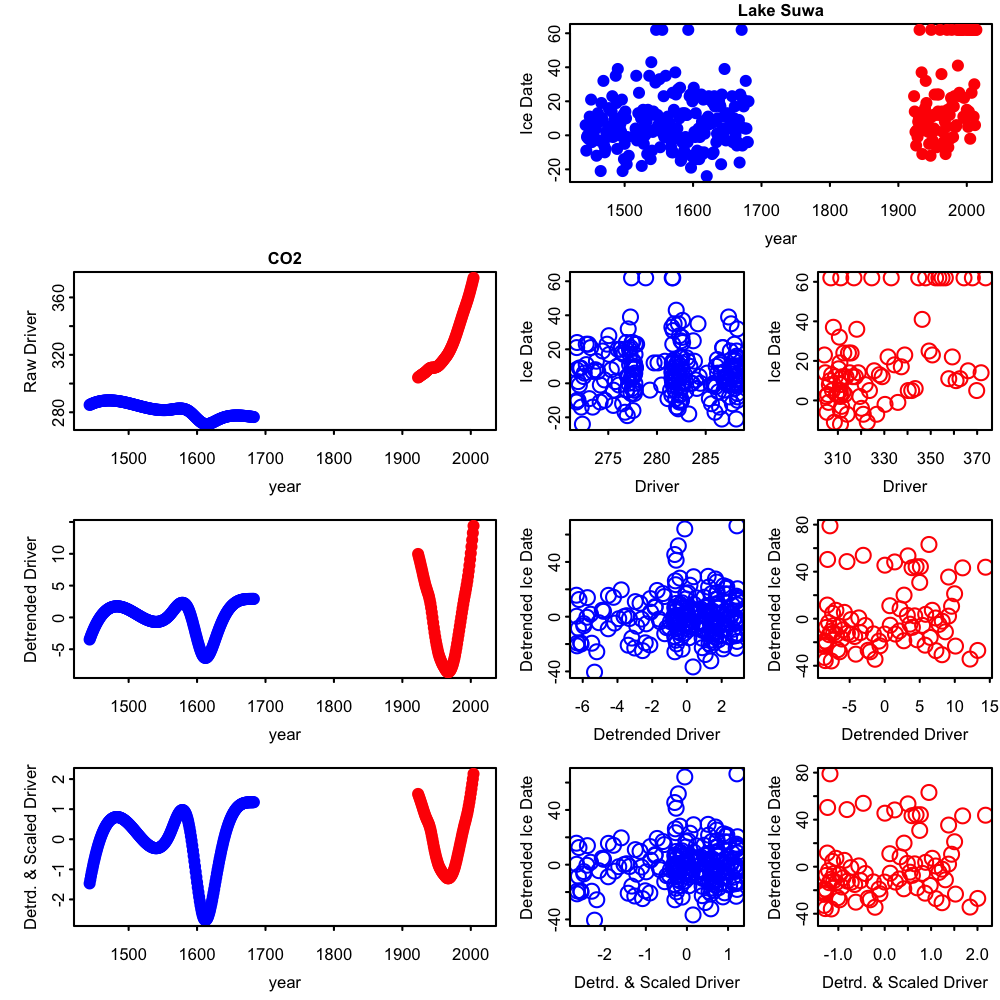


**Supplementary Fig. 3.** Time series of a driver (atmospheric CO_2_, ppm) and ice dates (Lake Suwa), and scatter plots of the two (driver on horizontal axis, ice date on vertical axis). Other conventions are as in Supplementary Fig. 2


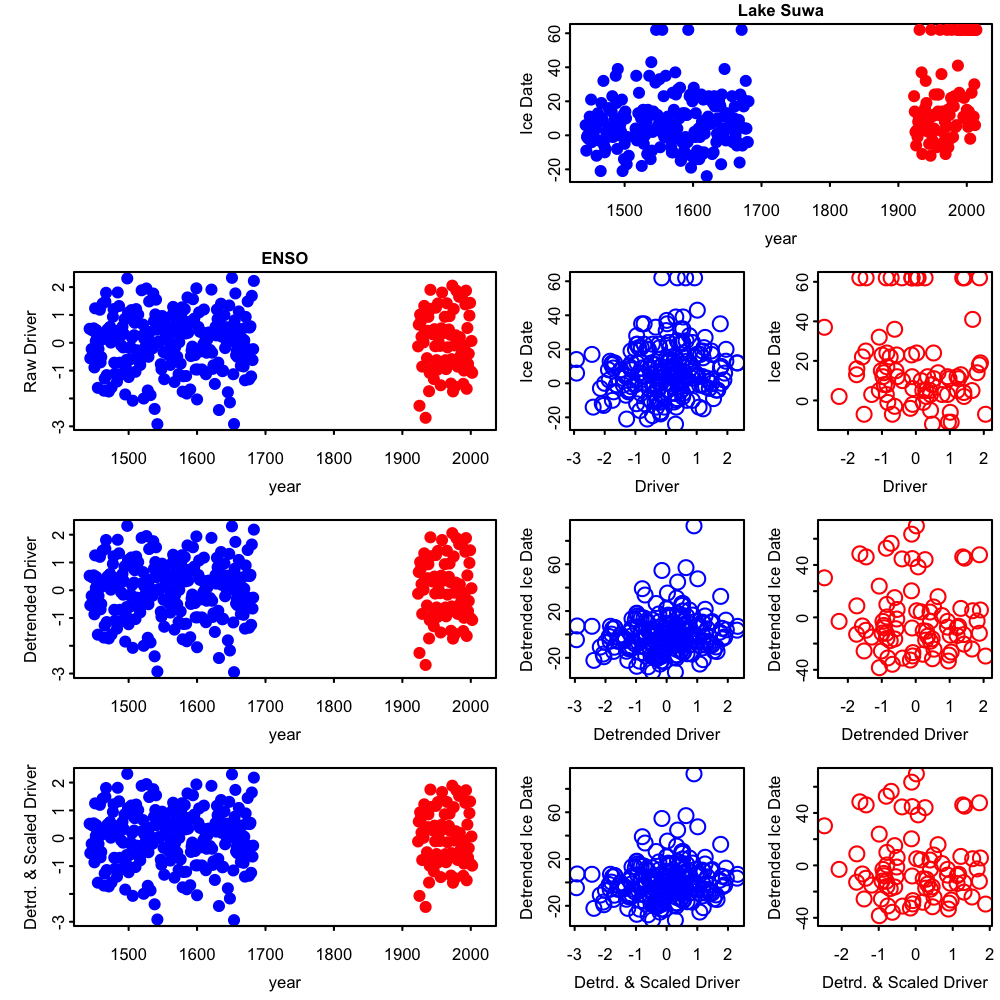


**Supplementary Fig. 4.** Time series of a driver (ENSO index) and ice dates (Lake Suwa), and scatter plots of the two (driver on horizontal axis, ice date on vertical axis). Other conventions are as in Supplementary Fig. 2.


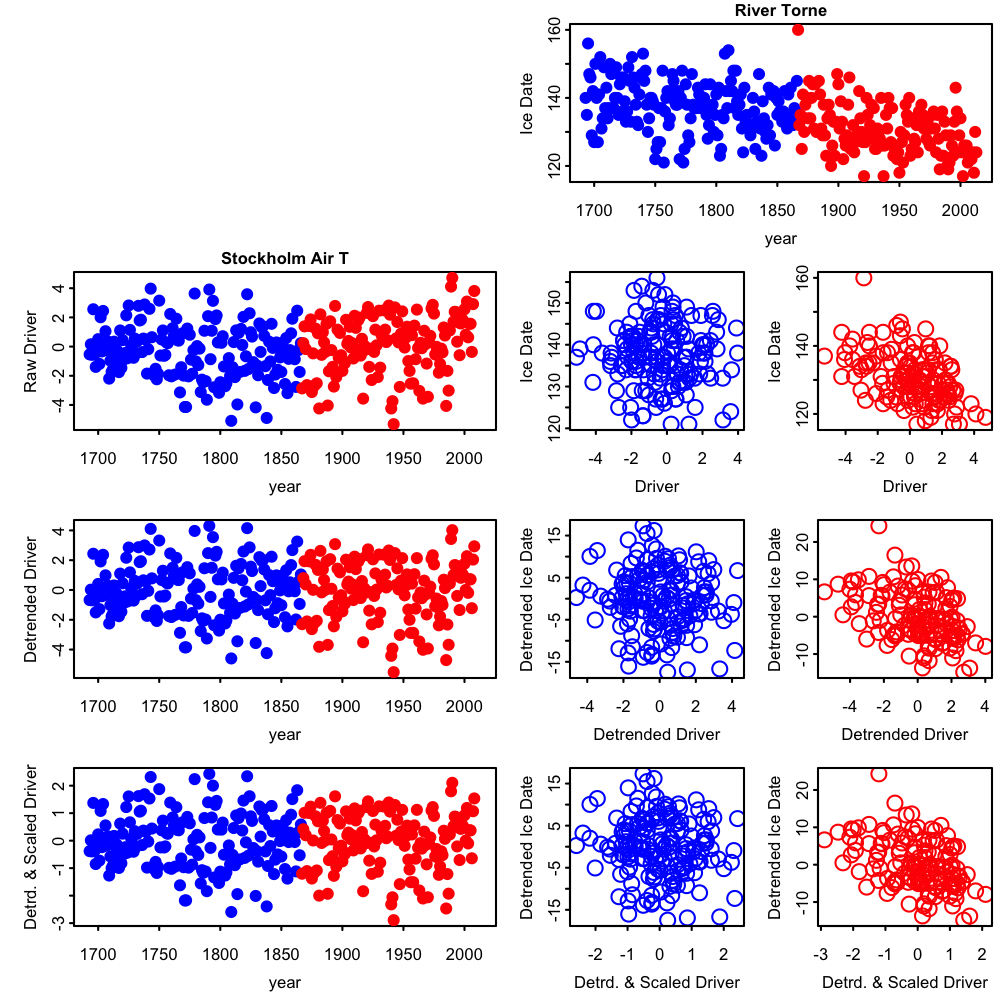


**Supplementary Fig. 5.** Time series of a driver (air temperature in Stockholm, Sweden, ºC) and ice dates (Torne), and scatter plots of the two (driver on horizontal axis, ice date on vertical axis). Other conventions are as in Supplementary Fig. 2.


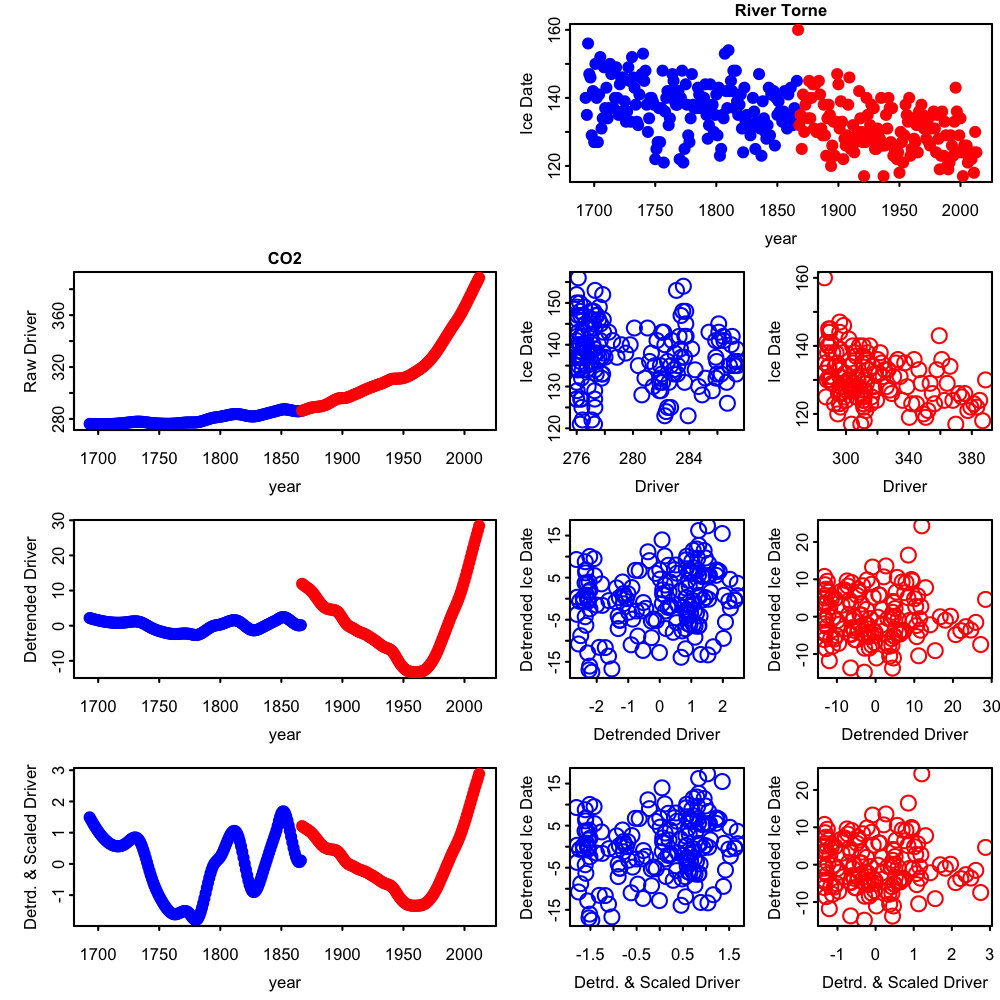


**Supplementary Fig. 6.** Time series of a driver (atmospheric CO_2_, ppm) and ice dates (Torne), and scatter plots of the two (driver on horizontal axis, ice date on vertical axis). Other conventions are as in Supplementary Fig. 2.


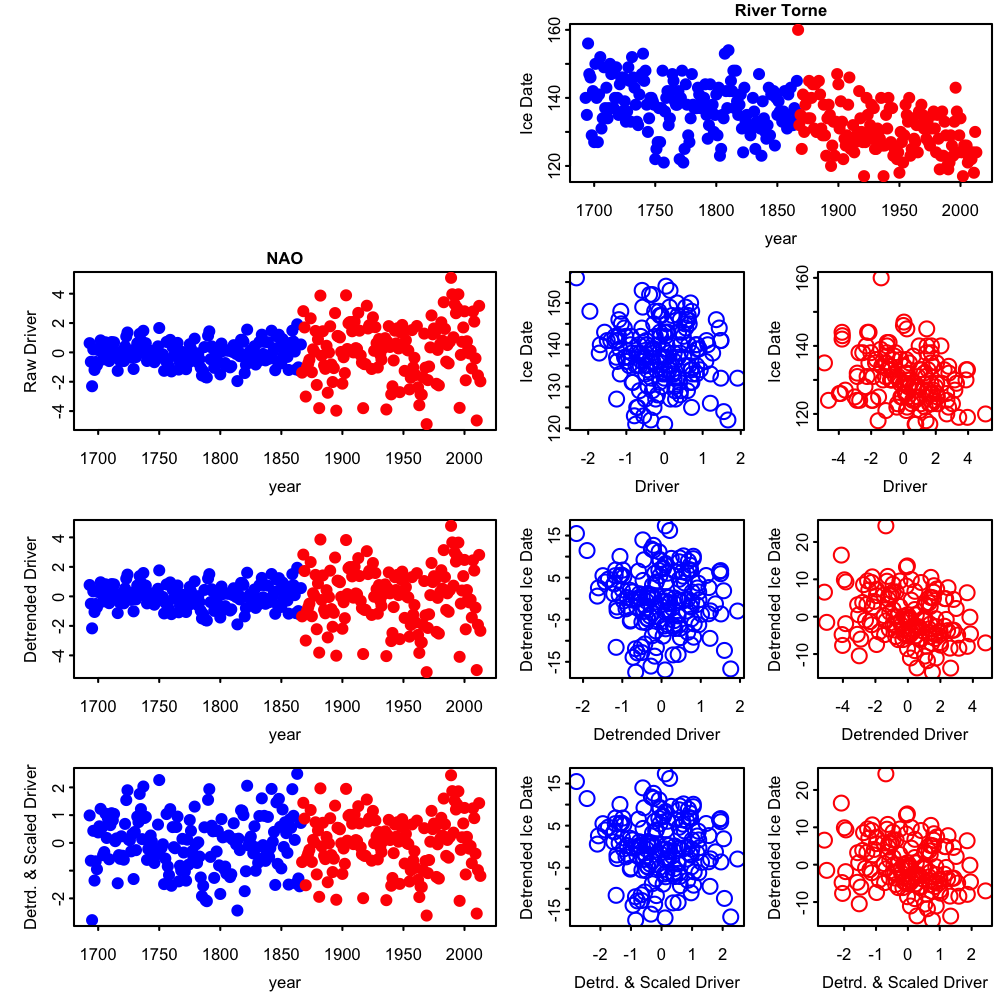


**Supplementary Fig. 7.** Time series of a driver (NAO index) and ice dates (Torne), and scatter plots of the two (driver on horizontal axis, ice date on vertical axis). Other conventions are as in Supplementary Fig. 2.


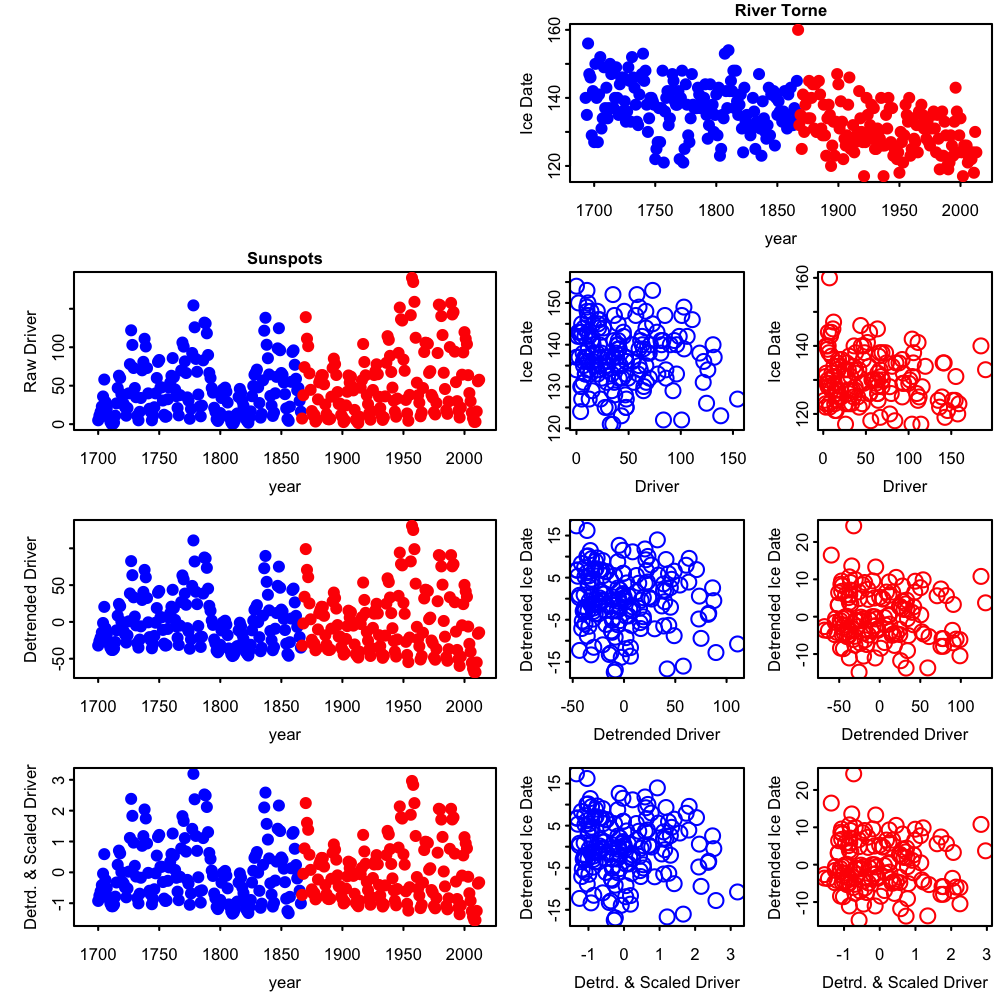


**Supplementary Fig. 8.** Time series of a driver (sunspot count) and ice dates (Torne), and scatter plots of the two (driver on horizontal axis, ice date on vertical axis). Other conventions are as in Supplementary Fig. 2

**Supplementary Table 1.** Summary of Suwa database based at the Snow and Ice Data Center. We excluded ice freeze dates from 1682-1923 from our analyses.

| **Years** | **Source code in Snow & Ice Data Center** | **Data available** | **Comments** |
| --- | --- | --- | --- |
| 1443-1993 | ARAI1 | 1443-1893 | From Fukiwhara in Arakawa (1954) and Yatsurugi Shrine (compiled and edited by T. Arai & Lake Ice Analysis Group) |
| 1897-1953 | ARAK1 | 1897-1953 | Arakawa (1954) |
| 1944-1993 | WSTA1 | 1944-1993 | Suwa Meteorological Observatory |
| 1899-2014 | YATSUI1 | 1989-2014 | Yatsurugi Shrine |

**Supplementary Table 2.**  List of major data sources for ice breakup dates of Torne River. For a complete description of every available data source and original commentary, please see Kajander (1995).

| **Years** | **Observer/Recorder in Torne (unless otherwise noted)** |
| --- | --- |
| 1693-1740 | Merchant Olof Ahlbom - with the exception of 1715-1721 when he escaped the Russian occupation |
| 1715-1721 | Anders Hellant |
| 1737-1749 | Johannes Wegelius and Anders Celcius |
| 1741-1787 | Anders Hellant |
| 1792-1837 | Parish clerk Johan Portin (in Overtonrea - 80 km north of Tornio) |
| 1830-1856 | Erik Burman (in Nederkalix - 45 km north of Tornio) |
| 1846-1855 | Finnish Society of Sciences |
| 1881-1894 | Finnish Society of Sciences |
| 1865-1879 | Meteorology journals housed at University of Helsinki Department of Meteorology |
| 1882 - present | Local newspapers, including Haparandabladet |
| 1898-1931 | Tornio newspapers |
| 1890-1910 | Meteorological Central Institute |
| 1882-present | Diaries and almanacs from private people |
| 1957-present | Ice breakup guessing competition |
| 1960-present | Finnish Environmental Institute and its predecessors (Hydrological Office) |

**Supplementary Table 3.** AIC values of fitted regression models relating ice date (y^*^*_i_*) to years elapsed (x_i_).

| Model | Torne AIC |
| --- | --- |
| y^*^*_i_* = β_0_ + β_1_*x_i_* + ε*_i_* | 2155.825 |
| y^*^*_i_* = β_0_ + β_1_*x_i_* +β_2_*x_i_*^2^ + ε*_i_* | 2154.881 |
| y^*^*_i_* = β_0_ + β_1_*x_i_* + β_2_max (*x_i_* – *a*_1_, 0) + ε*_i_* | 2153.072 |
| y^*^*_i_* = β_0_ + β_1_*x_i_* + β_2_max (*x_i_* – *a*_1_, 0) + β_3_ max(*x_i_* – *a*_2_, 0) + ε*_i_* | 2152.774^*^ |

^*^Breakpoints restricted to being at least 10 years apart; See Supplementary Fig. 1.
